# HRV Analysis: A Clinical and Diagnostic Tool in Chronic Obstructive Pulmonary Disease

**DOI:** 10.1155/2014/673232

**Published:** 2014-07-15

**Authors:** Aline Fernanda Barbosa Bernardo, Luiz Carlos M. Vanderlei, David M. Garner

**Affiliations:** ^1^Departamento de Fisioterapia, Universidade Estadual Paulista (UNESP), Presidente Prudente, SP, Brazil; ^2^Department of Biological and Medical Sciences, Faculty of Health and Life Sciences, Oxford Brookes University, Gipsy Lane, Oxford OX3 0BP, UK

## Abstract

This study's aim is to analyze heart rate dynamics in subjects with chronic obstructive pulmonary disease (COPD) by measures of heart rate variability (HRV). HRV is a simple and noninvasive measure of autonomic impulses. 38 adults were divided into two equal groups based on respiratory function: COPD and normal. HRV was monitored in the supine position for 30 minutes. After tests of normality, Kruskal-Wallis was used for the statistical analysis, with the level of significance set at *P* < 0.05. Principal component analysis identified two components representing 99.5% of total variance. Furthermore, it is suggested that the chaos forward parameter (CFP) which applies all three “chaotic globals” is the most influential, although others are statistically more significant. The COPD subjects exhibited a decrease in the CFP. COPD can be termed a dynamical condition, decreasing the chaotic response. The perceived benefits of such analysis include quantitative assessment and suitable pharmacological intervention in the respiratory condition, especially of other related dynamical diseases such as cardiac failure.

## 1. Introduction

Cardiac interbeat intervals fluctuate in a complex manner [1–4]. Time-series methods derived from statistical physics have motivated researchers to study this phenomenon [5]. The RR interval of the electrocardiograph (ECG) traces PQRST waveform is necessary for such computations. Heart rate variability (HRV) analysis using nonlinear dynamical techniques is becoming an important area of research. There is evidence that mechanisms involved in cardiovascular regulation interact with each other in a complex and a chaotic manner. This mathematical analysis of human illness is often termed “dynamical disease study [6].” Such algorithms are computationally processor intensive so they cannot be employed online, and are not effective on short time series. Compilation of data for such analysis usually requires observation for days or weeks [7]. Usually, changes in the HRV patterns are an indicator of health status. High HRV is a signal of good adaptation and characterizes a healthy person with efficient autonomic mechanisms. Whilst lower HRV is frequently an indicator of abnormal and insufficient adaptation of the autonomic nervous system, causing the subject low physiological function, this decrease is consistent with a dysfunctional vagus.

Detrended fluctuation analysis (DFA) [8] quantifies the presence or absence of fractal correlation properties of the consecutive heart beats. Applied to a number of dynamic phenomena, including HRV, fractal indices appear capable of detecting subtle changes in the dynamics of RR intervals better than conventional analyses.

Spectral entropy [9] and the new techniques, spectral detrended fluctuation analysis (sDFA) and spectral multitaper method (sMTM), are based on “chaotic globals” [10–12]. Briefly, spectral entropy applies the standard Shannon entropy [13, 14] algorithm to a power spectrum, whereas sDFA applies the DFA algorithm in the same manner to the same power spectrum. This attempts to overcome the disadvantage of sparse data hazard, only phase information is lost. sMTM applies the responsive and adaptive multitaper method (MTM) [15, 16] to the data. sMTM is the value of the area between the MTM spectrum and the baseline. We return to these parameters in Sections 3.1 to 3.3.

These computations are useful in monitoring surgical patients under anaesthesia [17, 18] or unable to communicate distress as in sleep apnea [19] or dyspnea [20–22]. Assessment of chaotic states in this way is both faster for diagnostic purposes and more efficient using less physician time, which is expensive. Here, the benefit is when assessing the risk of cardiac failure and other dynamical diseases in subjects with chronic obstructive pulmonary disease (COPD) [23]. The aim of the study is to develop an algorithm which can discriminate the datasets from subjects with COPD from those without COPD and thus provide a clinical and diagnostic tool to clinicians in the cardiopulmonary field.

## 2. Experimental Protocol

A total of 38 subjects were studied; 19 suffered COPD and 19 were deemed “normal” to be used as controls in this study. Data were collected under controlled temperature 21°C to 24°C and humidity 50% to 60%, and volunteers were instructed to avoid consuming alcohol and caffeine for 24 hours before evaluation. Data were collected between 8:00 and 11:00 to minimize the interference of circadian rhythm. All procedures necessary for the data collection were explained to the individuals, and the subjects were instructed to remain at rest and to avoid talking during the data collection.

After the initial evaluation, the heart monitor strap was placed on each subject's thorax over the distal third of the sternum. The HR receiver (Polar S810i monitor, Polar Electro OY, Kempele, Finland) was placed on the wrist. This equipment had been previously validated for beat-by-beat measurements and for HRV analysis. The subjects were placed in the supine position and remained at rest with spontaneous breathing for 30 minutes.

After the experimental procedures, spirometry was performed to confirm the diagnosis of COPD applying the forced vital capacity test pre- to postbronchodilator [24, 25] using a portable spirometer (MIR, Spirobank version 3.6, Italy) coupled to a microcomputer for analysis by WinspiroPRO 1.1.6 software. The forced expiratory volume in one second (FEV1) will be greater than or equal to 80% of the predicted normal values with an FEV1/FVC (forced vital capacity) that is less than 70%, which was considered as the threshold for bronchial obstruction [25].

HRV was recorded beat by beat through the monitoring process at a sampling rate of 1000 Hz. Exactly, 1000 RR intervals were used for analysis, following digital filtering complemented with manual filtering for the elimination of premature ectopic beats and artefacts. Only series with more than 95% sinus rhythm were included in the study.

## 3. Chaotic Global Parameters

Since the time series are short, we must apply power spectra to the data. Applying such algorithms to power spectra allows them to converge faster than computed on inter-peak temporal separations. Precision is increased for any fine detailed structure when we use Welch method [26] for spectral entropy [9] or sDFA. The sMTM applies the multitaper spectrum [15, 16]. In Sections 3.1 to 3.3, we summarize the chaotic global parameters.

### 3.1. Spectral Entropy

**Figure 1:**
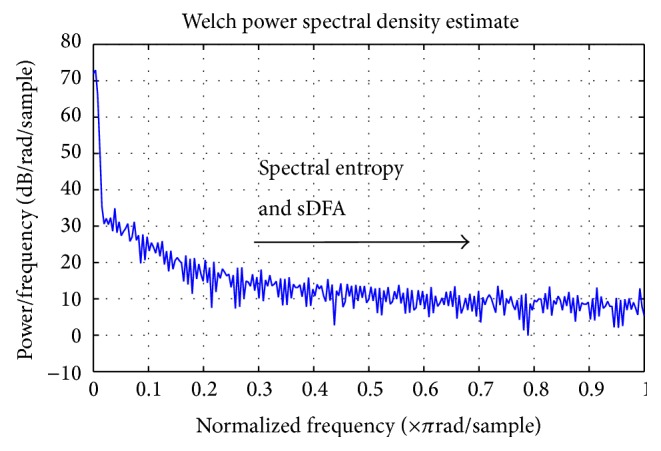
A Welch method power spectrum of a 1000 ECG RR intervals of a COPD patient.

Spectral entropy [9] is a function of the irregularity of amplitude and frequency of the power spectrums peaks. It is derived by applying Shannon entropy [13, 14] to power spectra. Here, we calculate the power spectrum by Welch's method [26] (see Figure 1). We set the parameters for the Welch power spectrum to sampling frequency of 1 Hz, zero overlap, a Hamming window with FFT length of 256, and no detrending.

This output is then normalized so that the sum of the magnitude is equal to unity, giving a normalized power spectrum. We then calculate an intermediate parameter which is the median Shannon entropy of the value obtained from three different power spectra using the Welch power spectra under three test conditions: a perfect sine wave, uniformly distributed random variables, and finally the experimental oscillating signal.

These values are then again normalized mathematically so that the sine wave gives a value of zero, uniformly random variables give unity, and the experimental signal gives a value between zero and unity. It is this final value that corresponds to spectral entropy.

### 3.2. Spectral Detrended Fluctuation Analysis

DFA [27, 28] can be applied to datasets where statistics such as mean, variance, and autocorrelation vary with time. The difference with the sDFA algorithm is that the DFA is applied to the frequency rather than time on the horizontal axis. So, once more, the *x*-axis is frequency and the *y*-axis is amplitude (see Figure 1). If the scaling exponent *α* in DFA is not constant for the duration of time for the dataset, such variability can introduce further errors even over short time periods (10–15 minutes). This reduces when power spectra are analyzed by DFA algorithm, but phase information is lost. To obtain sDFA we calculate the spectral adaptation in exactly the same way as for spectral entropy using a Welch power spectrum with the same settings, but DFA rather than Shannon entropy is the algorithm applied.

### 3.3. Spectral Multitaper Method

**Figure 2:**
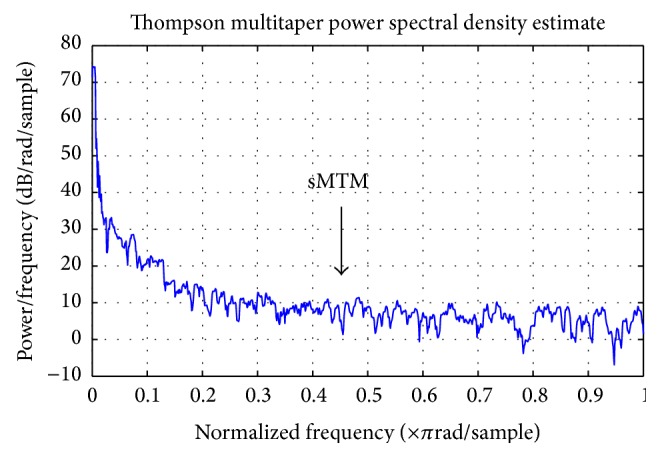
A multitaper method power spectrum of 1000 ECG RR intervals of a COPD patient.

sMTM is founded on the increased intensity of broadband noise in power spectra generated by irregular and chaotic signals. MTM provides estimates of both line components and the continuous background of the spectrum. MTM exploits the property that these adaptive orthogonally shaped windowed power spectra are extremely accurate. These optimal tapers belong to a family of spectral functions termed discrete prolate spheroidal sequences (DPSS) [29]. MTM spectral estimation reduces spectral leakage and other inaccuracies compared to the single windowed nonadaptive techniques. sMTM is the area between the MTM power spectrum and the baseline (see Figure 2). We set the parameters for MTM at sampling frequency of 1 Hz, time bandwidth for the DPSS set to 3, FFT length of 256, and Thomson's adaptive nonlinear combination method to combine individual spectral estimates.

### 3.4. Chaotic Forward Parameter

The parameter [CFP*x* 1–7] is referred to as chaotic forward parameter where it is applied to normal and COPD datasets. Since sDFA responds to chaos in the opposite way to the others, we subtract its value from unity when applying here. All three chaotic global values have equal weighting. [CFP*x* 1–7] are defined in the standard way as in Souza and Vanderlei [11, 12]. CFP1 is a function of all three parameters (spectral entropy, sMTM, and sDFA), CFP2 to CFP4 is a function of two (spectral entropy and sDFA: spectral entropy and sMTM; sMTM and sDFA), respectively. CFP5 to CFP7 is the function of a single chaotic global (sDFA; sMTM; spectral entropy), respectively.

## 4. Results

### 4.1. Statistical Analysis

**Figure 3:**
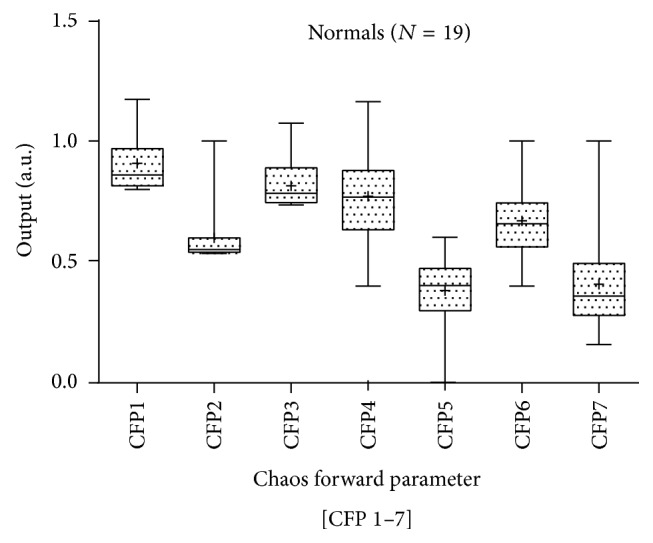
The boxplot illustrates the mean values and standard deviation of CFP for normal subjects RR intervals. The mean value is indicated by the (+) symbol in the boxplot.

**Figure 4:**
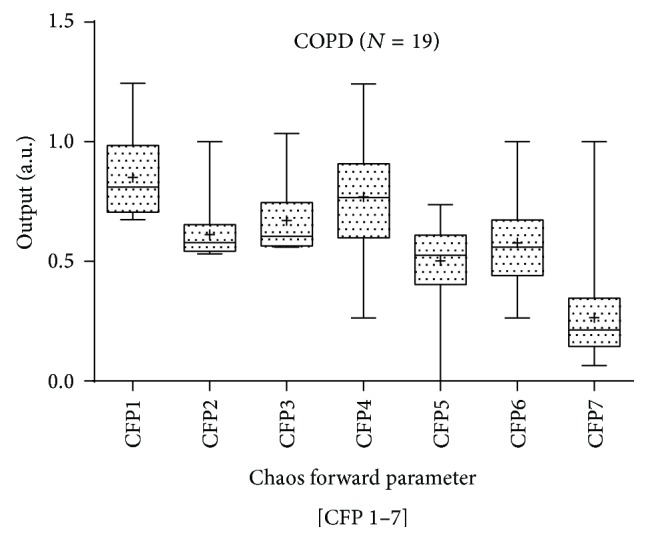
The boxplot illustrates the mean values and standard deviation of CFP for the RR intervals of COPD subjects. Mean values are indicated by the (+) symbol.

Parametric statistics generally assume the data are normally distributed and hence the use of the mean as a measure of central tendency. If we cannot normalize the data, we should not compare means. To test our assumptions of normality, we apply the Anderson-Darling [30] and Ryan-Joiner [31] tests. The Anderson-Darling test for normality applies an empirical cumulative distribution function, whereas the Ryan-Joiner test is a correlation based test. For both tests a normal distribution could not be confirmed; so we apply the Kruskal-Wallis [32] test of significance, a nonparametric test. The results illustrate that there is a wide variation in both the mean values and standard deviation for both cohorts (Figures 3 and 4). Only [CFP*x* 3, 5, & 7] are statistically significant at the level (*P* < 0.05) (see Table 1). However, whilst [CFP*x* 3 & 7] decreases from normal to diseased subjects, [CFP*x* 5] increases. See comparison between normals and COPD in Figures 3 and 4. Variation in standard deviations is minimal for [CFP*x* 1, 2, & 3]. It is also apparent that the interquartile ranges (Q3–Q1) of the COPD subjects are significantly higher than those of the normal subjects (see Table 1).

### 4.2. Principal Component Analysis

Principal component analysis (PCA) [33] can be applied here (See Table 2). We have the values of [CFP] for seven groups for 19 subjects who are suffering COPD; hence a grid of 7 by 19 is to be assessed. The first principal component has a variance (eigenvalue) of 4.1588 and accounts for 59.4% of the total variance. The second principal component has an eigenvalue of 2.8078 accounting for 99.5% of total variance. Therefore, we can assume that most variance is achieved in the first two components.

Now, only [CFP*x* 3, 5 & 7] are significantly different when tested by Kruskal-Wallis. When assessing the importance of the results by PCA, [CFP*x* 1], which applies all three chaotic globals techniques, is the best overall combination with regard to influencing the correct outcome. [CFP*x* 7], which is just spectral entropy, is the next best. [CFP*x* 3] is the third greatest which omits the sDFA function.

Therefore, [CFP*x* 1 & 3] are the most suitable functions as deduced by the three assessments (Kruskal-Wallis, standard deviation, and principal component analysis). [CFP*x* 3] would seem to outperform [CFP*x* 1] on the basis of this study, but there is evidence to apply [CFP*x* 1] as the most robust function, as in the paper which analyzes the “inverse problem” posed by Garner and Ling [10]. This is in addition to forward problems in obesity [11] and diabetes mellitus [12].

## 5. Discussion

The mathematical analysis is undertaken such that it is not only appropriate for online analysis but also retrospective in the laboratory and clinical setting. Here, the analysis is done retrospectively for the time series for each [CFP*x*], applied to normal and COPD datasets for the ECG's RR intervals. The algorithm computes a significant statistical result for three of the seven combinations. These are combinations 3, 5, and 7.

Conversely, the first algorithm which applies all three chaotic globals parameters is suggested as the most robust algorithm. Referring to Garner and Ling [10], who use three models, Duffing, Brusselator, and Lorenz, for the purposes of optimization, [CTF] a variant of [CFP*x*] is the most reliable objective function when tested by PCA. This is reinforced here by PCA applied to the seven different versions of [CFP] for subjects with COPD. Here, 99.5% of influence is achieved by the first two principal components, with the [CFP] with all three chaotic globals applied testing as most influential algorithm.

Increased statistical significance is achieved by [CFP*x* 3], derived from the spectral entropy and sMTM alone. However, this combination is only the second most appropriate when assessed by PCA. [CFP*x* 5] and [CFP*x* 7] are significant too and the interpretation of the PCA values suggests they are also high-ranking overall. In fact, [CFP*x* 7] is only outperformed on PCA by [CFP*x* 1].

Further improvement for future study could involve modification of the Welch power spectra for the sDFA and spectral entropy. The sampling frequency, extent of overlap, and detrending could be attuned. A higher spectral resolution technique such as the MTM may prove beneficial. The DPSS of the MTM could be adjusted to optimize the final level of significance by its *P* value. In addition, the weighting of the three chaotic global parameters could be adjusted since here they have only equal weightings of unity. It would also be statistically favourable to have larger datasets for both normal and COPD.

## 6. Conclusions

We have developed two robust functions [CFP*x* 1 & 3] which can take short-times series of HRV and deduce which time series is from a COPD patient and which time series is from the normal subjects. There is a high level of significance for the [CFP*x* 3] algorithm (*P* < 0.001). Nevertheless, the algorithm which applies all three parameters [CFP*x* 1] is the most influential when assessed by PCA. By applying either of these novel functions to the shorter time series via spectrally determined “chaotic globals,” it should be possible to determine which time series are COPD or normal, more rapidly and efficiently with regard to time and data length. There has been a significant decrease in chaotic response measured by [CFP*x* 1 & 3] of HRV in COPD. The relationship between COPD and complexity measures is useful as a diagnostic tool. It identifies severity of the respiratory condition from a cheap and reliable method of monitoring the autonomic nervous system through ECG and RR intervals. This is helpful in treatments, such as determination of the level of pharmacological intervention especially in related dynamical diseases such as cardiac failure.

## Figures and Tables

**Table 1:** The table below shows the first (Q1) and third (Q3) quartiles of [CFP*x* 1–7] for the normal and COPD subjects in 1000 RR intervals. The statistical significance Kruskal-Wallis test is applied. A nonparametric test.

[CFP*x*]	Normal Q1	Normal Q3	COPD Q1	COPD Q3	Kruskal-Wallis
1	0.8130	0.9680	0.7060	0.9841	0.1116
2	0.5362	0.5953	0.5421	0.6530	0.3502
3	0.7427	0.8877	0.5648	0.7458	0.0005
4	0.6300	0.8769	0.5977	0.9079	0.9651
5	0.2919	0.4683	0.4034	0.6088	0.0072
6	0.5583	0.7414	0.4412	0.6735	0.0749
7	0.2721	0.4898	0.1448	0.3451	0.0066

**Table 2:** The table below is the principal component analysis for CFP for seven groups for 19 subjects who are suffering from COPD. PC1 represents the first principal component, PC2 the second, until the seventh component PC7.

Variable	PC1	PC2	PC3	PC4	PC5	PC6	PC7
CFP1	0.357	−0.409	0.105	−0.639	0.202	−0.141	−0.474
CFP2	0.045	−0.590	0.660	0.248	0.017	0.272	0.281
CFP3	0.197	−0.544	−0.501	0.496	0.002	−0.408	−0.041
CFP4	0.486	0.075	−0.028	−0.320	−0.211	−0.294	0.724
CFP5	0.448	0.239	0.312	0.282	−0.619	−0.105	−0.413
CFP6	0.489	−0.007	−0.385	0.050	0.042	0.780	0.009
CFP7	−0.394	−0.355	−0.237	−0.315	−0.727	0.186	0.017
